# Worldwide surveys on anaphylaxis to sublingual immunotherapy with house dust mite tablets are urgently needed

**DOI:** 10.1002/clt2.12012

**Published:** 2021-03-29

**Authors:** Ralph Mösges, Desiderio Passali, Mario Di Gioacchino

**Affiliations:** ^1^ IMSB (Institute of Computational Biology and Medical Statistics) University at Cologne Cologne 50924 Germany; ^2^ Clinical Research International Limited Mühlenberg 64 Hamburg 22587 Germany; ^3^ Istituto di Discipline Otorinolarinogologiche Universita degli Studi Siena 53100 Italy; ^4^ Department of Medicine and Science of Ageing G. d'Annunzio University Chieti‐Pescara Chieti 66100 Italy; ^5^ Institute for Clinical Immunotherapy and Advanced Biological Treatments Pescara 65100 Italy

## Abstract

In the 1980s, a global number of 72 fatalities were reported in the UK and the USA following the application of subcutaneous immunotherapy (SCIT). This resulted in a significant limitation of SCIT use and in the search of other routes of administration, among which sublingual immunotherapy (SLIT) showed the best balance between efficacy and safety. Data from controlled studies suggest that tablets‐related anaphylaxis is an uncommon event. However, in the Eudravigilance (European database of suspected adverse drug reactions from Europe) we found reports of life‐threatening events or severe local reactions under SLIT increasing over the last few years. Therefore, all efforts to minimize the related risk have to be strongly encouraged.

To the Editor,

The issue of fatal reactions to subcutaneous immunotherapy (SCIT) in patients with respiratory allergy emerged in the 1980s, when a global number of 72 fatalities were reported in the United Kingdom and the United States of America.^1^ This resulted in a significant limitation of SCIT use and in the search of other routes of administration, among which sublingual immunotherapy (SLIT) showed the best balance between efficacy and safety. Indeed, the identification of concomitant uncontrolled asthma at the time of the allergen extract injection as the major risk factor for fatalities was a key advance in the prevention of severe adverse reactions. Actually, avoiding the allergen injection in patients with a current forced expiratory volume in one second (FEV1) under 70% of predicted value, the measures to prevent administration mistakes and the prompt availability of rescue equipment in a supervised setting, contributed to making fatal reactions very rare. As to SLIT, the first systematic review on its safety found that local reactions in the site of contact with the allergen, that is the oral cavity and the gastrointestinal system, were quite common, while systemic reactions were rare.^2^ Of interest, there was no significant difference in the safety profile comparing studies on low dose products used in the early trials with those based on higher doses as suggested in the Allergic Rhinitis and its Impact on Asthma(ARIA) document, which highlighted that doses at least 50 times higher than SCIT were needed to predict clinical efficacy.^3^ In three decades of SLIT use, there have been rare case reports of SLIT‐associated anaphylaxis, estimated in about one case per 100 million administrations.^4^ However, the latest generation SLIT tablets, which contain high doses of grass pollen native extract seem concerned by increasing reports of suspected anaphylactic reactions. Recently, dust mite tablets were developed and, based on evidence of efficacy, tolerability, and safety of application were included in the 2017 update of the Global Initiative on Asthma guideline as add‐on therapy in mite allergic adult patients who have asthma exacerbations despite ICS treatment, provided a FEV1 value of at least 70% of predicted is measured. Nolte et al.^5^ recently analyzed the occurrence of reactions requiring epinephrine in 8152 actively treated patients in trials with the three high dose SLIT tablets, including 1‐grass pollen (*Phleum pretense*) (13 trials), ragweed (five trials), and dust mites (11 trials): an overall number of 16 tablet‐related epinephrine administrations (eight for grass pollen, four for ragweed pollen, and four for dust mite tablets) were identified (0.2% of subjects), of which six for systemic reactions and 10 for severe local reactions. None of the events were reported as serious, or with airways impairment, but some included chest discomfort, cough, dyspnea or were described as anaphylactic, not excluding that the further progression of the reaction could have been interrupted by the epinephrine administration. Remarkably, five administrations occurred after the first week of treatment (the latest at Day 128), prompting an Food and Drug Administration mandate for the prescription and use training of epinephrine autoinjectors for patients on SLIT, being unable to receive medical care in a timely manner outside of the clinical setting.^6^ Although this precaution is a matter of debate in the United States,^7^ the anecdotal late occurrence of anaphylactic reaction, even months from treatment beginning, was reported also in Europe. On the other hand, it is not a standard practice outside of the United States to prescribe autoinjectors for SLIT patients. This may however become mandatory, if data presented recently at the German Allergy Conference should be confirmed: in this real‐world study 25 *serious* adverse drug reactions were reported in 1525 patients treated with HDM‐tablets during the first year. Most of these events occurred during the self‐administration of tablets outside of the practices.^8^

This data suggests the need of a careful postmarketing surveillance and signal detection to monitor the risk of severe systemic reactions. The report from the EudraVigilance (European database of suspected adverse drug reactions from Europe), concerning anaphylaxis to SLIT was further worrying. Actually, from 2016 to December 2019 there were 82 reports of suspected anaphylaxis to mite products (15 concerning pediatric patients and six defined as anaphylactic shock), 54 of them related to native allergen tablets without buildup phase.^9^ The number was progressive year by year corresponding to the spread of these products in the market (6 in 2016, 13 in 2017, 24 in 2018, and 39 in 2019; Figure 1). Notably, eight cases occurred from the second to the fifth, nine from the sixth to the tenth, two from the tenth to the twentieth, one beyond the 20th day of treatment. The remaining 28 reports were referred to other preparations, as oral solutions or drops or unbranded oral lyophilizate, none to modified allergen tablets and drops.

**FIGURE 1:**
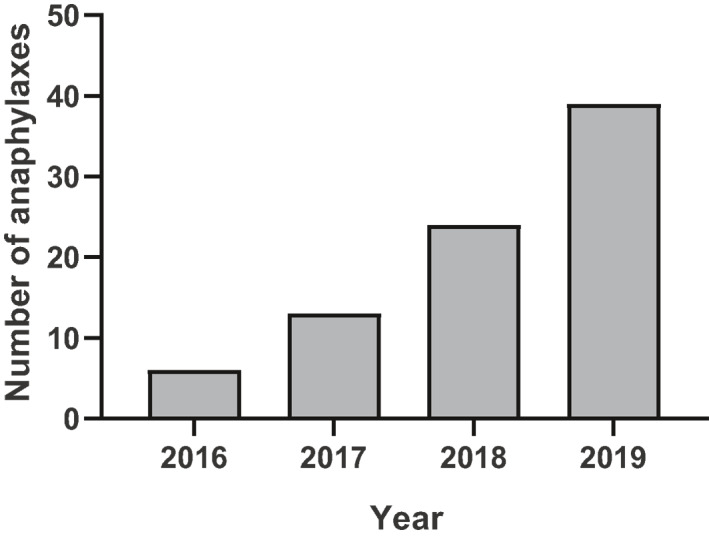
Numbers of reported suspected cases of anaphylaxis during house dust mite SLIT per year in the EMA repository WWW.ADRreports.EU. EMA, European Medicines Agency; SLIT, sublingual immunotherapy

Looking at these data, both from controlled studies and from the real routine practice pharmacovigilance database, it is clear that tablets‐related anaphylaxis remains an uncommon event. However, the risk of life‐threatening events or severe local reactions appears often underestimated. Despite the clinical criteria for diagnosing anaphylaxis, statistics based on spontaneous reports is a known controversial issue and the occurrence of epinephrine administration in placebo‐treated patients is a further confounding aspect. However, the risk of severe reactions is actually perceived and contemplated in the US practice parameter.^10^ Therefore, specific studies are urgently needed to explore the risk factors associated to SLIT‐related anaphylaxis, in order to anticipate and prevent the occurrence of fatalities. In particular, the possible role of the biologic potency of the allergen extracts, the schedules of administration, and the favoring role of concomitant conditions or, especially in adults, comorbidities warrant to be investigated.

Respiratory allergy (asthma and rhinitis) can occur with different severity and severity‐related treatments are currently available. AIT is of particular importance in this context being the only causal treatment of allergy. Thus, in a view of a risk‐benefit ratio, even single cases of life‐threatening events in our opinion may once again raise questions about the justifiability of AIT for allergic diseases of lesser clinical relevance and all efforts to minimize the related risks should be strongly encouraged.

## CONFLICT OF INTERESTS

Prof. Mösges reports personal fees from Allergopharma, grants and personal fees from Allergy Therapeutics, grants and personal fees from Bencard, grants and personal fees from Leti, grants and personal fees from Lofarma, personal fees from ALK, grants and personal fees from Stallergenes, grants from Inmunotek, personal fees from HAL, grants from Aimmune, during the conduct of the study; grants from Optima, personal fees from Friulchem, grants and personal fees from Hexal, grants and personal fees from Klosterfrau, personal fees from FAES, personal fees from Meda, personal fees from Novartis, personal fees from UCB, grants and personal fees from BitopAG, grants from Hulka, grants from Ursapharm, personal fees and nonfinancial support from Menarini, personal fees from Mundipharma, personal fees from Pohl‐Boskamp, personal fees from Hikma, personal fees from Sandoz, grants and personal fees from Lek, grants and personal fees from Cassella, personal fees from SanofiGenzyme, personal fees from Engelhard, nonfinancial support from SmartPeakFlow, personal fees from Strathos, outside the submitted work; and Ralph Mösges is the director and the owner of Clinical Research International Ltd., and of ClinCompetence Cologne GmbH, two contract research organizations focusing on upper airways diseases. Prof. Di Gioacchino reports grants and personal fees from GSK, grants from Novartis, grants, personal fees and nonfinancial support from Lofarma, personal fees from Menarini, grants from SanofiGenzyme, outside the submitted work. Prof. Passali reports personal grants from Lofarma, personal grants from DMG, outside the submitted work;

## AUTHOR CONTRIBUTIONS

Ralph Mösges conducted the data base search, drafted, and finalized the manuscript. Desiderio Passali reviewed the manuscript and contributed his own opinion statements. Mario Di Gioacchino reviewed the manuscript and contributed his own opinion statements. All authors read and approved the final manuscript.
